# Obesity, Dietary Patterns, and Cardiovascular Disease: A Narrative Review of Metabolic and Molecular Pathways

**DOI:** 10.3390/cimb47060440

**Published:** 2025-06-10

**Authors:** Cristina Dina, Delia Mirela Tit, Ada Radu, Gabriela Bungau, Andrei-Flavius Radu

**Affiliations:** 1 Doctoral School of Biological and Biomedical Sciences, University of Oradea, 410087 Oradea, Romania; cristina.dina@nutricareclinics.ro (C.D.); gbungau@uoradea.ro (G.B.); andreiflavius.radu@uoradea.ro (A.-F.R.); 2 Department of Pharmacy, Faculty of Medicine and Pharmacy, University of Oradea, 410028 Oradea, Romania; 3 Department of Psycho-Neurosciences and Recovery, Faculty of Medicine and Pharmacy, University of Oradea, 410073 Oradea, Romania

**Keywords:** cardiovascular diseases, obesity, dietary interventions, nutritional status, risk factors

## Abstract

Cardiovascular diseases (CVDs) remain the leading global cause of death, with obesity acting as a significant contributing factor through mechanisms such as chronic inflammation, insulin resistance, and endothelial dysfunction. Molecular pathways at the interface of obesity, diet, and CVDs reveal how altered lipid metabolism, oxidative stress, and inflammatory signaling contribute to CVD progression. Despite advancements in treatment, effective management of CVDs, particularly in the context of obesity, remains a challenge. This review addresses the gap in understanding the relationship between obesity, nutritional status, and CVD progression, evaluating the impact of dietary interventions such as low-carb, Mediterranean, ketogenic, and Dietary Approaches to Stop Hypertension diets on cardiovascular health. Key findings indicate that adipokines, interleukins, and tumor necrosis factor alpha play significant roles in inflammatory responses and insulin resistance, further exacerbating cardiovascular dysfunction. Furthermore, optimized dietary strategies have been shown to modulate several molecular pathways, improving cardiovascular risk factors and enhancing metabolic health. This review underscores the significance of understanding molecular metabolic pathways in the intricate relationship between obesity, diet, and CVDs. It highlights the role of personalized nutrition and comprehensive dietary patterns in the management of CVDs and advocates for further research to optimize dietary strategies for sustained cardiovascular health.

## 1. Introduction

Cardiovascular diseases (CVDs) represent the leading cause of death and disability worldwide [1]. In 2021, ischemic heart disease was identified as the primary contributor to global mortality, which accounted for 9.44 million deaths, followed by ischemic stroke, intracerebral hemorrhage, and hypertensive heart disease. The most significant risk factors with major implications for health include high systolic blood pressure, unhealthy dietary patterns (i.e., high intake of sodium, sugar, and saturated fats, combined with low intake of fruits, vegetables, and fiber-rich foods), high low-density lipoprotein cholesterol, particulate matter pollution, smoking, long fasting periods, plasma glucose, and body mass index (BMI) [2,3].

Obesity, a key risk factor that is associated with insulin resistance, diabetes, hypertension, cardiac arrhythmias, heart failure, and coronary artery disease, plays a substantial role in the development of CVDs, primarily through mechanisms such as chronic inflammation, endothelial dysfunction, and metabolic disturbances. This underscores the need for integrated public health strategies that address both obesity and its underlying pathophysiological mechanisms to improve cardiovascular outcomes [4]. Excess visceral adiposity has been identified as an independent predictor of poor cardiovascular outcomes by studies that measure fat depots, including ectopic fat [5].

Insulin resistance promotes dyslipidemia, while obesity-related systemic and vascular inflammation increases the risk of low-density lipoprotein cholesterol (LDL) oxidation. These mechanisms contribute to atherogenesis, independent of other established risk factors such as hypertension and hyperglycemia [6].

Atrial fibrillation (AF) and sudden cardiac death are more prevalent in individuals with obesity, not only due to structural changes in the heart but also because of chronic systemic inflammation and autonomic dysfunction. Furthermore, obesity contributes to heart failure through hemodynamic changes, increased blood pressure, cardiac fibrosis, and left ventricular dysfunction, ultimately leading to ventricular dilation and hypertrophy [7].

Gradual intensification of care approaches is the foundation of obesity management. The two categories of treatment are pharmacological (i.e., drug therapy and surgical management) and nonpharmacological (i.e., behavioral lifestyle adjustment). Dietary and physical-activity-related lifestyle recommendations are advised throughout the early phases of treatment [8].

Poor diet quality is strongly associated with elevated risk of CVD morbidity and mortality. Evidence-based dietary guidelines for cardiometabolic health emphasize balanced energy intake, including a variety of fruits and vegetables, whole grains, lean protein sources, plant oils, and minimal consumption of processed foods, added sugars, and salt. They also recommend limited alcohol intake and adherence to these principles regardless of food preparation or consumption settings [9].

Extensive research has demonstrated that both the Dietary Approaches to Stop Hypertension (DASH) [10,11] and Mediterranean (MD) [12,13] diets are linked to improved cardiovascular outcomes. These dietary patterns help reduce the incidence of cardiovascular disease by enhancing weight management and reducing low-grade inflammation, which, in turn, positively affects other cardiovascular risk factors [14]. Therefore, optimizing nutritional status becomes critical in this context for effectively managing cardiovascular disease progression and enhancing patient outcomes.

The present paper aims to present evidence on the relationship between nutritional status, particularly obesity and overweight, and cardiovascular disease progression. By evaluating the effectiveness of various dietary interventions, such as low-carb, low-calorie, Mediterranean, ketogenic, and DASH diets, and examining the role of personalized nutrition, this review seeks to offer novel insights into innovative strategies for weight management and cardiovascular disease prevention and improvement, thereby bridging gaps in current knowledge and clinical practice.

## 2. Obesity and Overweight

The worldwide increase in obesity rates seems to be predominantly influenced by changes in the food system, which currently generates greater volumes of processed, heavily advertised, and economically accessible food items compared to previous years [15]. The excessive intake of energy resulting in obesity is an expected consequence of capitalist economies that rely on development determined by consumption. The promoters of the worldwide food system interact with local environmental aspects to generate considerable disparities in obesity prevalence across different populations. The difference in body size across individuals within communities can be attributed to the connections involving individual and environmental aspects, which include genetic features. Regardless of the existence of personal heterogeneity, this medical issue exhibits consistent patterns within specific subpopulations. Obesity mainly impacts middle-aged individuals, specifically women, from affluent metropolitan regions in low-income states. In contrast, in high-income countries, obesity impacts individuals regardless of age or gender, yet it is prevailing among deprived populations [16].

The prevalence rate of obesity, adapted for age, has risen from 4.6% in 1980 to 14.0% in 2019. The incidence of obesity peaks in the American and European regions, with Russia and the United States having the highest amount of obese individuals [17]. According to research on body composition, both men and women reach their fat mass maxima around the midpoint of their middle years. However, after this age, the percentage of body fat keeps going up, specifically in men due to a far increased loss of lean mass [18].

Globally, pediatric obesity has become more widespread in the last 50 years. The incidence of obesity in young people between 5 and 19 years increased significantly for girls, from 0.7% to 5.6%, and for boys, from 0.9% to 7.8%, between 1975 and 2016 [19]. Moreover, the World Obesity Federation predicted that by 2030 about 254 million young people aged 5–19 years will be impacted by obesity [20].

Obese individuals (e.g., those with a BMI of 30 or more) present an increased risk of developing coronary heart disease (CHD) and facing increased mortality rates, in contrast to those with a normal weight. The failure of BMI to take into consideration the repartition of body fat is a major shortcoming of the metric. Measures applied to assess the way body fat is distributed take into account the waist-to-hip ratio (WHR), waist-to-height ratio (WHtR), and waist circumference (WC) [21].

### 2.1. Obesity-Associated Complications

The transition from a lean to an obese state induces alterations in adipose tissue phenotype and elicits a state of chronic low-grade inflammation. This phenomenon is characterized by increased levels of soluble pro-inflammatory markers such as interleukin (IL)-6 and -1β, tumor necrosis factor (TNF) α, and monocyte chemoattractant protein (MCP) 1, along with circulating free fatty acids and the infiltration and activation of immune cells in inflammatory regions [22]. Low-grade chronic inflammation plays a pivotal role in the pathogenesis and progression of various severe conditions, including malignancies, CVD, and hepatic disorders [23,24].

The prevalence of CVD is alarming, owing to its substantial impact on death rates, contribution to reduced labor capacity, and burden on healthcare systems. Although there have been advancements in treatment, CVD continues to be the primary cause of death among people suffering from obesity, responsible for 70% of all mortalities in this population [25].

Several mechanisms contribute to the negative consequences of obesity for the heart and blood vessels. As obesity advances, inflammatory cell infiltration occurs in the pancreas, adipose tissue, and various other organs. The immune system’s cells infiltrate unhealthy adipose tissue, setting off an inflammatory cascade. Coronary calcification is induced during the atherosclerotic inflammatory phase, which is linked to obesity. It can be identified as a negative influence on cardiac remodeling, determined by chamber enlargement, hypertrophy, and impaired ventricular diastolic and systolic function. Furthermore, high blood pressure and high leptin levels are associated with obesity. Leptin activates the sympathetic nervous system and influences nitric oxide (NO) generation, leading to salt retention, systemic vasoconstriction, and elevated blood pressure. For both adults and children, obesity increases the likelihood of developing CVD and other serious health problems, including metabolic syndrome, heart failure, high blood pressure, AF, dyslipidemia, and depression [26].

The etiology of vascular endothelial cell dysfunction and atherosclerosis is significantly influenced by oxidative stress resulting from raised levels of circulating saturated fatty acids. Oxidative stress plays a key role in the development of metabolic disorders and degenerative processes [27]. The buildup of harmful fatty acids, such as ceramides and diacylglycerides, leads to dysfunction of mitochondria, stress in the endoplasmic reticulum, and the production of reactive oxygen species. The aforementioned factors collectively cause insulin resistance, inflammation, the onset of atherosclerosis, and apoptosis of endothelial cells [28].

A comprehensive analysis of more than 300,000 individuals with 18,000 coronary artery disease (CAD) events indicated that an elevated risk of CAD is associated with BMI in the overweight and obese populations. Age ranges varied between studies, with most cohorts having baseline age ranges between 40 and 60 years. For example, the Nurses’ Health Study included women aged 34–59, while the US Railroad Cohort included adults aged 40–59 at baseline. Several cohorts were sex-specific: for example, the Nurses’ Health Study included only women, whereas the Health Professionals Follow-up Study, Italian Rural Areas Study, PRIME Study, Rome Railroad Cohort, US Railroad Cohort, Whitehall Study, and Zutphen Elderly Study included only men. Physical activity and smoking were adjusted for in the analysis, but comorbidities such as diabetes and hypertension were not consistently reported or adjusted for. Data on menopausal status were not mentioned, despite its known relevance to weight regulation in women [29].

Obesity was associated with a lower coronary volume-to-myocardial mass ratio, suggesting that the structure and function of the heart in obese individuals may be compromised, potentially leading to other cardiovascular problems, even if obstructive coronary stenosis was less evident [30]. Moreover, obesity markedly elevates the risk of cardiovascular illnesses via a confluence of direct and indirect processes, encompassing inflammation, insulin resistance, hypertension, dyslipidemia, and structural alterations to the heart. Despite the lack of significant coronary stenosis, obesity continues to be a significant factor in cardiovascular health problems. Addressing obesity by lifestyle modifications, dietary adjustments, and physical activity is essential for mitigating the risk of cardiovascular disease.

#### 2.1.1. Chronic Inflammation

In the context of obesity, chronic low-grade inflammation and the progression of atherosclerosis are driven by the altered release of adipose-derived hormones such as leptin, resistin, and adiponectin, accompanied by elevated circulating levels of proinflammatory mediators, including TNF-α, IL-6, and MCP-1, as well as an increase in systemic aldosterone concentrations [31].

Leptin initiates its intracellular signaling cascade upon autophosphorylation of its receptor (LepR), subsequently engaging multiple downstream molecular pathways. These include the activation of Janus kinases (JAKs), which in turn stimulate signal transducer and activator of transcription (STAT) proteins, along with additional signaling routes involving insulin receptor substrate (IRS) and phosphatidylinositol 3-kinase (PI3K), adenosine monophosphate-activated protein kinase (AMPK), extracellular signal-regulated kinases (ERKs), and mitogen-activated protein kinases (MAPKs) [32].

Increased leptin concentrations can serve as a marker for leptin resistance. This resistance can arise from factors such as genetic alterations, leptin’s autoregulatory mechanisms, limited access to tissues, and the molecular and cellular control of circulation [33].

Leptin produced within the organism may contribute to cardiac hypertrophy by engaging various mechanisms, including rapamycin pathway activation, calcineurin and NFAT stimulation, MAPK 14 (p38) activation, and elevated levels of intracellular reactive oxygen species [32]. Leptin-induced aldosterone secretion represents a new mechanism driving endothelial dysfunction and cardiac fibrosis in obesity, which disrupts myocardial relaxation and exacerbates CVD [34].

Resistin, mainly secreted from macrophages in humans, is described as a hormone linking obesity to type 2 diabetes mellitus, a disorder marked by dysregulated glycemia due to impaired insulin dynamics and glucagon excess [35,36]. Circulating levels of resistin positively correlate with obesity, promoting both inflammation and insulin resistance [35]. Resistin influences the synthesis of critical pro-inflammatory cytokines, including TNFα, IL6, and IL12, by activating NF-κB pathways in macrophages, leading to significant disruptions in peripheral insulin signaling and the development of insulin resistance [37,38].

Resistin interacts with TLR4 in the hypothalamus, triggering the recruitment of adaptor proteins MyD88 and TIRAP, which leads to the activation of downstream signaling pathways. This activation of the resistin/TLR4 pathway disrupts hypothalamic insulin sensitivity by modifying insulin receptor (IR), AKT, and ERK1/2 phosphorylation. Such effects may be due to resistin-induced downregulation of IR and increased expression of suppressor of cytokine signaling 3 (SOCS3) and protein tyrosine phosphatase 1B (PTP1B) in the hypothalamus, both of which are critical inhibitors of insulin signaling [35]. Furthermore, SOCS3 acts as a key negative regulator of insulin and leptin signaling. In obesity, its hypothalamic upregulation impairs insulin receptor- and STAT3-mediated leptin pathways, particularly in proopiomelanocortin neurons. This contributes to hypothalamic insulin and leptin resistance, promoting energy imbalance, glucose intolerance, and weight gain [39,40]. PTP1B impairs hypothalamic insulin and leptin signaling and is upregulated in obesity, where it contributes to central insulin resistance. In adipose tissue, PTP1B negatively regulates adipocyte differentiation by inhibiting adipogenic transcription factors, such as PPARγ and SREBP-1c, and mediates TNFα-induced adipose dysfunction, promoting chronic inflammation and lipid overflow [41,42].

Adiponectin, one of the key adipokines, plays a protective role in processes like energy regulation, inflammation, and cellular growth. It has been shown to protect against several inflammatory conditions, including atherosclerosis, cardiovascular diseases, and insulin resistance, primarily through the activation of the AMPK and cAMP-PKA pathways. The molecular actions of adiponectin may involve direct effects on inflammatory cells, reducing reactive oxygen species, promoting the production of the anti-inflammatory cytokine IL-10, inhibiting the NF-κB signaling pathway, and decreasing inflammatory responses related to TNF-α [43].

An increase in adipocyte size may lead to a shift from M2 to M1 macrophages, triggering the release of leptin, angiotensin, reactive oxygen species, and pro-inflammatory cytokines like IL-6, IL-1, and TNF-α [22]. TNF-α is involved in various aspects of the pathophysiological processes underlying obesity, diabetes, and CVDs [44].

Cardiac methylation is directly influenced by the upregulation of DNMT1. In the presence of TNF-α, cardiomyocytes show a threefold increase in *ATP2A2* gene promoter methylation, resulting in reduced protein expression and impaired calcium regulation. Such methylation alterations are implicated in the pathophysiology of heart failure (HF). Additionally, the activation of the renin–angiotensin system (RAS) elevates oxidative stress, while increased TNF-α levels in circulation further promote DNA hypermethylation in HF [45].

IL-6 plays a critical role in metabolic and vascular functions, significantly contributing to the development of insulin resistance, type 2 diabetes, and atherosclerosis. It impairs hepatic insulin sensitivity, reduces insulin-dependent glycogen synthesis, and limits glucose uptake by adipocytes, while also decreasing liver glycogen storage. These changes result in lower plasma insulin levels and elevated glucose levels, particularly in animal models, leading to hyperglycemia and hyperlipidemia. Additionally, IL-6 increases circulating levels of free fatty acids, C-reactive protein (CRP), and reactive oxygen species (ROS), while suppressing nitric oxide (NO) production. These effects play a key role in endothelial dysfunction and lipoprotein oxidation, which are central mechanisms in the pathogenesis of atherosclerosis [46].

Monocyte Chemoattractant Protein-1 (MCP-1) is central to inflammation triggered by obesity, acting by attracting monocytes to adipose tissue through its receptor, CCR2. Elevated levels of MCP-1 are found in the visceral fat of individuals with obesity, which lead to an influx of macrophages and sustained, low-grade inflammation. This inflammatory process plays a significant role in the development of insulin resistance, independent of the amount of body fat, as demonstrated by studies in humans and animals. Furthermore, MCP-1 supports the formation of new adipocytes via MCPIP and fosters angiogenesis within the adipose tissue, promoting its expansion. Diet, such as fructose consumption or low-glycemic food intake, and hormones like vitamin D and parathyroid hormone, influence circulating MCP-1 levels. Inhibiting the MCP-1/CCR2 pathway has emerged as a potential strategy for alleviating insulin resistance and inflammation in adipose tissue, showing promise for treating metabolic disorders linked to obesity [47].

#### 2.1.2. Oxidative Stress

Oxidative stress plays an essential role in the onset of various obesity-related conditions, such as hypertension and dyslipidemia, both of which contribute significantly to the development of CVDs. The accumulation of reactive oxygen species (ROS) induces lipid modifications, resulting in the formation of oxidized low-density lipoprotein (Ox-LDL), a key factor further exacerbating CVD. Ox-LDL has a substantial impact on adipokine production, which may, in turn, initiate additional oxidative stress. In individuals with obesity and dyslipidemia, the increase in Ox-LDL levels can be attributed to a reduction in the antioxidant defense mechanisms, primarily due to the decreased activity of enzymes like superoxide dismutase (SOD) and paraoxonase-1 (PON-1) associated with HDL. PON-1 is integral to the protective effects of HDL, offering anti-inflammatory, antioxidant, and anti-atherogenic properties. Furthermore, elevated levels of Ox-LDL may arise from a higher oxidative potential, such as the enhanced expression of NADPH oxidase 2 (NOX2), which further reduces adiponectin synthesis, increases the secretion of pro-inflammatory cytokines, and generates more ROS in the vascular and immune cells circulating throughout the bloodstream [48].

Emerging studies highlight the pivotal involvement of NADPH-mediated ROS production and the activation of redox-sensitive signaling pathways in the pathophysiology of hypertension induced by Angiotensin II (Ang II) [49]. The interaction between Ang II and its Ang-II type 1 receptor (AT1r) triggers the activation of NADPH oxidase in nonphagocytic cells, leading to the production of reactive oxygen species, including superoxide, hydrogen peroxide (H_2_O_2_), and peroxynitrite. In addition, factors such as sodium retention and the heightened activation of the sympathetic nervous system (SNS), driven by elevated insulin levels, may further stimulate the renin–angiotensin–aldosterone system (RAAS), which sustains the development of hypertension [50].

Recent data showed the presence of additional molecular mediators (i.e., adiponectin, ghrelin, and leptin) and neuropeptides like neuropeptide Y and α-melanocyte-stimulating hormone, along with inflammatory cytokines (i.e., IL-1, IL-6, and TNF-α), that functionally bridge adipose tissue dysregulation, oxidative stress, and elevated blood pressure [51].

#### 2.1.3. Insulin Resistance

CVDs are frequently accompanied by conditions such as insulin resistance, dyslipidemia, and excess body fat. In the context of obesity-related diabetes, the pattern recognition receptor Dectin-1 has emerged as a pivotal mediator of adipose tissue inflammation. Its expression is markedly increased in individuals with obesity and type 2 diabetes, where it shows a positive association with both glycated hemoglobin (HbA1c) levels and inflammatory biomarkers like TNF-α. At the molecular level, sustained hyperglycemia and elevated TNF-α stimulate Dectin-1 transcription through NF-κB interaction with its promoter sequence. Once activated, Dectin-1 intensifies local inflammation by engaging signaling cascades such as Syk-CARD9, IRF5, and NF-κB, ultimately fostering chemokine-dependent infiltration of immune cells into adipose depots [44].

Insulin resistance involves lipid-driven and nutrient-sensing signaling disruptions. Elevated free fatty acids in obesity promote ectopic lipid storage, activating atypical PKC isoforms that disrupt insulin receptor signaling, impairing glucose uptake in muscle and reducing hepatic glycogen synthesis. Additionally, mTOR and S6K1 activation in nutrient excess leads to serine phosphorylation of IRS-1, reducing PI3K activity and dampening insulin signaling. In the heart, insulin resistance results in damage through three key mechanisms: altered signaling, impaired substrate metabolism regulation, and reduced nutrient delivery to myocardial tissue [52].

## 3. Dietary Interventions in Patients with Weight Disorders

### 3.1. Low-Fat Diet

The ratio of calories obtained from fat on a low-fat diet ranges from 20 to 25%, whereas on an extremely low-fat diet it drops to 10 to 20% [53]. The latest recommendations of the National Cholesterol Education Program for adults indicate keeping saturated fat intake below 7% of total calories and cholesterol below 200 mg daily. These recommendations are based on the results of the Adult Treatment Panel III (ATP III). Based on the guidelines, polyunsaturated fats should not exceed 10% of total calories, whereas monounsaturated fats are recommended to account for no more than 20% [54].

A link exists between obesity and microvascular dysfunction. The effects of a low-fat diet on microvascular function, aside from weight loss, remain ambiguous; yet this diet does mitigate cardiovascular risk. Mahmoud et al. evaluated low-fat weight maintenance (LFWM) diets in comparison with low-fat weight loss (LFWL) diets to assess their effects on NO-mediated vasodilation in microvessels. Over a six-week period, eleven individuals with obesity received the LFWL diet, while ten followed the LFWM diet. To assess vascular reactivity to acetylcholine (ACh) and flow, microvessels were isolated from gluteal subcutaneous fat biopsies collected both before and after dietary intervention, with and without indomethacin or Nω-nitro-l-arginine methyl ester (L-NAME). Additional measurements included serum and vascular NO as well as C-reactive protein (CRP) levels. The results showed a significant enhancement in acetylcholine-induced dilation (AChID) and flow-induced dilation (FID) in response to the low-fat, whole-food weight loss diet. This effect was abolished by L-NAME, indicating an NO-dependent mechanism. In contrast, the LFWM diet did not affect AChID or FID. Both diets reduced the initial enhancement of FID and AChID observed with indomethacin. However, neither intervention influenced serum NO or CRP levels. These findings suggest that the LFWL diet improves microvascular function via increased NO bioavailability, whereas the LFWM diet does not exert similar vascular benefits. This randomized intervention included 21 obese adults (BMI: 30–39.9 kg/m^2^), aged 18–50, with a majority of females (14 out of 21). Postmenopausal and perimenopausal women were excluded, ensuring premenopausal female participants. All had no comorbidities, such as diabetes, hypertension, or CVD. Physical activity was kept at habitual levels and monitored via pedometers, with no significant differences in step counts between the groups. The study’s design allowed the isolation of dietary effects from age, sex, and comorbidity influences [55].

Yang et al. evaluated two macronutrient dietary patterns, the low-carbohydrate, high-fat (LCHF) diet and the low-fat, high-carbohydrate (LFHC) diet, to determine which was more effective for weight loss and cardiovascular risk reduction. Their meta-analysis revealed that the LCHF diet produced a more significant reduction in body weight and greater increases in HDL cholesterol compared to the LFHC diet. However, changes in total cholesterol and LDL cholesterol were less pronounced between the two dietary approaches. No significant differences were observed in blood pressure (both systolic and diastolic), glucose levels, triglycerides, lean mass, or fat mass between groups. These findings suggest that while LCHF diets may offer advantages in weight loss and HDL cholesterol improvement, their effects on other cardiovascular risk markers may be comparable to those of LFHC diets. This meta-analysis included 11 Randomized Controlled Trials (RCTs) with 739 overweight or obese adult participants aged between 40 and 54 years. Most studies enrolled more women than men, and one trial included only female participants; however, postmenopausal status was an exclusion criterion, and menopausal effects were not analyzed. Physical activity and comorbidities were not assessed or reported as part of the included studies. Participants did not have pre-existing cardiovascular conditions or risk factors. Therefore, age and gender were partially addressed, but physical activity and comorbidity influences remained unreported in the analyzed trials [56].

### 3.2. Low-Carbohydrate Diet

A low-carbohydrate diet (LCD) is characterized by consuming fewer carbs than the recommended range for healthy individuals, which is typically 45–65% of overall daily energy consumption. This diet often involves consuming between 50 and 130 g of carbohydrates per day or 10–45% of overall calorie consumption [57]. Currently, most LCDs primarily utilize protein as an energy source instead of lipids, helping to mitigate the negative effects of abnormal lipid metabolism without the need for costly protein supplements, while also supporting the preservation of muscle mass. However, individuals with obesity are at a higher risk of developing kidney problems, and excessive protein intake may further exacerbate renal health issues, making it crucial to monitor protein consumption in this population [58].

Chawla et al. critically examined dietary and lifestyle interventions in the context of the obesity pandemic through a meta-analysis comparing the effects of LCDs and low-fat diets on weight, HDL cholesterol, LDL cholesterol, triglycerides, and total cholesterol in adults. The analysis showed that after 6 to 12 months, LCDs provided greater benefits in terms of average weight loss (mean difference: −1.30 kg; 95% CI: −2.02 to −0.57), HDL cholesterol (increase of 0.05 mmol/L; 95% CI: 0.03 to 0.08), and triglyceride levels (decrease of −0.10 mmol/L; 95% CI: −0.16 to −0.04), whereas low-fat diets demonstrated more favorable effects on LDL cholesterol and total cholesterol. Overall, the meta-analysis concluded that LCDs positively influence weight reduction as well as lipid profiles related to HDL cholesterol and triglycerides. The RCTs included for evaluation comprised 6499 adult participants aged between 33 and 58 years, with BMIs ranging from 22 to 43.6 kg/m^2^. While studies involving major comorbidities were excluded, some trials did include individuals with obesity-related risk factors. Physical activity was prescribed in a subset of studies, either as general advice or in structured programs. Gender was not consistently stratified in the outcome reporting, and data on menopausal status were not specified, thus limiting conclusions on gender-specific responses, particularly in postmenopausal women [59].

Silverii et al. conducted a comprehensive meta-analysis of 25 randomized controlled trials assessing the effects of LCDs on BMI and cardiovascular risk factors in obese individuals. Across the 25 trials evaluated (N ≈ 2442), the mean participant age ranged from ~27 to 68 years, with most studies reporting values in the 40s–50s. Females were included in all studies, with five trials enrolling only women. Menopausal status was not reported. Physical activity was not systematically described across studies. Regarding comorbidities, seven studies did not report data regarding diabetes mellitus, nine studies excluded diabetic individuals, and nine included them. Moreover, 12 excluded participants with cardiovascular diseases, and 6 excluded those with chronic kidney disease. The analysis found that LCDs were associated with a significantly lower BMI at 3–4 and 6–8 months compared to control diets, whereas no differences were observed at 10–14 and 18–30 months. A greater reduction in BMI was particularly notable at 3–4 months, with no significant changes at later follow-up periods. No statistically significant changes in fasting plasma glucose were detected at any time point. Although TGs decreased and HDL increased at 18–30 months, no significant long-term changes were found in blood pressure, total cholesterol, or LDL. While LCDs demonstrate significant short-term benefits for weight loss and some cardiovascular risk factors, further research is required to clarify their long-term efficacy and renal safety before they can be recommended as the optimal dietary approach for obese patients. The impact of LCDs on renal function and overall well-being could not be fully evaluated, as only four studies reported data on renal function, as described in the next paragraph [60].

A 12-month randomized trial comparing low-carbohydrate (<40 g/day) and low-fat (<30% fat) diets found that the low-carbohydrate group experienced greater weight loss (mean difference: −3.5 kg [95% CI: −5.6 to −1.4 kg]; *p* < 0.001) and reductions in fat mass (−1.5% [CI: −2.6% to −0.4%]; *p* = 0.011) compared to the low-fat group. Additionally, the low-carbohydrate diet led to a larger decrease in the total to HDL cholesterol ratio (−0.44 [CI: −0.71 to −0.16]; *p* = 0.002) and triglyceride levels (−0.16 mmol/L [−14.1 mg/dL]; *p* = 0.038), along with a greater increase in HDL cholesterol (+0.18 mmol/L [7.0 mg/dL]; *p* < 0.001). Both diets achieved similar reductions in LDL cholesterol. Serum creatinine showed moderate decreases without significant differences between groups [61]. The second study showed that there were no significant differences in renal function between high-protein (HPD) and high-carbohydrate (HCD) low-fat diets over one year in morbidly obese patients. Creatinine levels remained stable, showing minimal change (HPD: +0.03 mg/dL; HCD: +0.02 mg/dL), with values staying within healthy ranges. Both diets, combined with cognitive behavior therapy, had no adverse impact on renal function, indicating that macronutrient composition (protein vs. carbohydrate) does not affect kidney health during weight loss [62]. The third study found that a low intake of both carbohydrates and lipids did not adversely affect renal function, as indicated by consistent creatinine levels. Concerns of renal strain from ketogenic diets (KDs) were not substantiated [63]. Finally, the fourth study found no significant changes in creatinine, blood urea nitrogen, or albumin-to-creatinine ratios after 4 months in patients on a diet low in carbohydrates and lipids, indicating a stable renal function [64].

A comprehensive study of RCTs examined the impact of low-fat diets (≤30% of energy from fat) and low-carbohydrate diets (LCDs; ≤45% of energy from carbohydrates) on metabolic risk factors. Among the 23 included RCTs (N = 2788), most enrolled overweight or obese adults had a mean age ranging from 30 to 50. Gender was variably addressed: five trials enrolled only women. Menopausal status was not specified. Physical activity was not systematically reported. Most trials excluded participants with diabetes, cardiovascular disease, or chronic kidney disease. Both low-carbohydrate and low-fat diets were effective in reducing weight and improving metabolic risk variables. Individuals on low-carbohydrate diets experienced slightly smaller reductions in total cholesterol and LDL compared to those on low-fat diets, but larger increases in HDL and smaller reductions in triglycerides. The two diets were statistically indistinguishable in terms of reducing metabolic risk factors, including body weight and WC [65]. Another analysis included 12 randomized studies targeting the clinical implications of LCDs. The included trials enrolled adults aged 31 to 65 years, with an average age ranging from approximately 40 to 58 years across studies. The gender distribution was reported, with a predominance of female participants in most trials; however, menopausal status was not assessed. None of the included studies provided details regarding participants’ physical activity levels or the presence of comorbidities, which represent important confounding factors potentially influencing weight loss and cardiovascular outcomes. These studies showed that a low-carbohydrate diet (LCD) was associated with a reduction in triglyceride concentrations of −0.15 mmol/L. A reduction of −0.23 mmol/L was observed for LCDs lasting 6 months or less, while a decrease of −0.17 mmol/L was linked to LCDs lasting 12–23 months. In the observation groups, participants’ body weights decreased by 1.58 kg; with an intervention duration of 6–11 months, this decrease was 1.14 kg, and with a duration of less than 6 months, it was 1.73 kg. In the control group, systolic blood pressure declined by 1.41 mm Hg, diastolic blood pressure by 1.71 mm Hg, plasma HDL levels by 0.1 mmol/L, and serum total cholesterol by 0.13 mmol/L. Fasting blood glucose levels changed by 0.03 mmol/L, and plasma LDL levels increased by 0.11 mmol/L, neither of which values were statistically significant. Further investigation is needed to assess the long-term effects of LCDs on cardiovascular risk factors, but this meta-analysis demonstrates that these diets have a positive impact [66].

### 3.3. Low-Calorie Diet

The “calories in–calories out” model underpins many eating plans. When a diet is solely based on creating a negative energy balance, the body’s adaptive mechanisms counteract weight reduction efforts by decreasing energy expenditure. A low-calorie diet typically involves a daily intake ranging from 1000 to 1500 calories, while an intake of 800 calories or fewer is classified as an extremely low-calorie diet. To achieve a negative energy balance, low-calorie diets generally restrict carbohydrates or fats. However, over the long term, such diets may not be sustainable. Severely reduced caloric intake should not be recommended without prior consultation with a healthcare provider. It has been suggested that combining behavioral treatments with a very-low-calorie diet enhances weight loss outcomes more effectively than behavioral interventions alone [67].

A medical study assessed the value of plasma biomarkers and determined whether weight loss benefits the cardiovascular system, even if diabetes is not remitted. Twenty-nine individuals with type 2 diabetes were examined both before and after diet-induced weight loss. Remission from diabetes, weight loss, QRISK (10-year cardiovascular risk), the duration of diabetes, and lipid-related markers were analyzed to evaluate changes in plasma adipokines and lipid-related indicators. After weight loss, QRISK decreased significantly in both responders and non-responders, although non-responders remained at a higher risk. A longer duration of diabetes was associated with higher QRISK and plasma GDF-15 levels at baseline. In both groups, GDF-15, leptin, and FGF-21 levels decreased, while adiponectin levels increased following weight loss. However, in non-responders, FGF-21 levels remained elevated. Changes in plasma VLDL1-TGs were associated with shifts in QRISK. As HDL cholesterol levels increased, leptin levels decreased, and adiponectin levels rose, showing a favorable correlation. Ultimately, weight loss significantly reduced cardiometabolic risk, particularly after diabetes remission. The decrease in body fat was associated with a decrease in plasma leptin, FGF-21, and GDF-15 levels, and an increase in adiponectin levels, regardless of diabetes remission status. Following substantial dietary weight loss and remission of type 2 diabetes, it is feasible to normalize the 10-year cardiovascular disease risk and heart age [68].

Moderate calorie restriction (an approximately 12% reduction) over two years in healthy, non-obese young and middle-aged adults significantly improved various cardiometabolic risk markers despite modest weight loss (~7.5 kg). The intervention led to notable reductions in LDL cholesterol, the total cholesterol-to-HDL ratio, systolic and diastolic blood pressure, and inflammatory marker C-reactive protein levels. Furthermore, insulin sensitivity and metabolic syndrome scores improved substantially compared to controls. These favorable changes persisted independently of the degree of weight loss, indicating that calorie restriction itself exerts beneficial effects on cardiovascular risk factors beyond simple weight reduction. Thus, even in the absence of obesity, sustained moderate calorie restriction may provide meaningful cardiometabolic advantages and holds promise for long-term cardiovascular health improvement in the general population [69].

Caloric restriction (CR) reduces visceral fat and systemic inflammation, with improvements observed across metabolic syndrome and aging populations. In obese women, CR lowered heart rate, glucose, insulin, and visceral adiposity, directly contributing to cardiovascular protection. Animal studies corroborate these findings mechanistically, linking CR to downregulation of angiotensin-converting enzyme and AT1 receptors, components of the renin–angiotensin system that mediate hypertensive and fibrotic responses. Furthermore, intermittent fasting regimens demonstrate protective effects against myocardial injury in preclinical models, with reductions in cardiac remodeling and enhanced survival. While evidence regarding severe CR is more mixed, highlighting risks such as arrhythmias or natriuretic peptide elevations, short-term interventions (e.g., 800 kcal/day) in obese patients have still yielded measurable improvements in metabolic syndrome scores and cardiovascular risk factors. Collectively, current data offer clear and converging support for the cardioprotective role of mild to moderate CR, especially when applied under clinical supervision and sustained over time [70].

Intermittent fasting combined with CR (IFCR), particularly when paired with liquid meals, has demonstrated notable reductions in body weight, visceral fat, and several cardiovascular risk markers in obese women over 10 weeks. An IFCR–liquid group experienced greater decreases in LDL cholesterol (20%) and triglycerides, alongside an increase in LDL particle size, indicating improved lipid profiles linked to reduced CHD risk. These lipid improvements correlated with decreases in pro-atherogenic adipokines such as leptin, IL-6, and TNF-alpha, which are implicated in vascular inflammation and dyslipidemia. The observed benefits appear to be driven by the greater caloric deficit and visceral fat loss, highlighting the importance of fat distribution beyond total weight loss. Although promising, findings are limited to female subjects and short-term interventions, underscoring the need for more comprehensive studies to validate long-term cardiovascular benefits of IFCR [71].

Scientific evidence indicates that low-calorie, high-protein intermittent fasting (HP-IF) diets may offer measurable cardiovascular and weight-related benefits. In a controlled trial over 64 weeks with 40 obese adults, a 12-week HP-IF intervention resulted in significant reductions in body weight, BMI, and triglyceride levels (~25%), alongside modest LDL decreases (~11%). Arterial compliance improved post-intervention and remained more stable after one year in the HP-IF group compared to a heart-healthy diet group. Although blood pressure and heart rate increased slightly with some weight regain, baseline values remained improved relative to pre-intervention levels. Importantly, reductions in arterial stiffness in the HP-IF group suggest sustained vascular benefits. While the results are promising, sample size and adherence limitations temper broad generalization. Nevertheless, this trial provides direct physiological evidence that targeted CR with protein emphasis and intermittent fasting may help maintain long-term cardiometabolic improvements in obese adults. The study enrolled 40 middle-aged obese adults (mean age: 48.0 ± 1.4 years; males: 46.1 ± 1.5, females: 50.0 ± 2.3), all non-smokers and either sedentary or lightly active (<30 min, 2 days/week). Participants had no cardiovascular or metabolic diseases and were not receiving hormone therapy. While both sexes were included (21 males, 19 females), the menopausal status of female participants was not reported. Comorbidities were controlled through exclusion criteria rather than analyzed variables, and physical activity remained minimal throughout the trial [72].

A recent meta-analysis of RCTs evaluated the cardiovascular effects of CR in adults, primarily those with overweight or obesity. The included RCTs enrolled adult participants with a weighted average age of 48–49 years. While most studies included individuals with overweight or obesity (average BMI: 31.6 kg/m^2^), data on physical activity levels and comorbidities, apart from baseline hypertension (reported in 13% of studies), were generally not described. The findings showed that short-term interventions (1–4 weeks) reduced systolic and diastolic blood pressure by approximately 5.5 mmHg and 2.9 mmHg, respectively, effects comparable to antihypertensive drugs. Medium-duration interventions (up to 6 months) sustained these benefits and additionally lowered the resting heart rate. Mechanistically, these effects may be mediated by reduced sympathetic nerve activity and improved endothelial function. However, most interventions involved strict or rapid caloric deficits, limiting long-term feasibility. While CR improved cardiorespiratory fitness when combined with exercise, its isolated benefits on cardiovascular function were modest and tightly linked to weight loss. Overall, this meta-analysis suggests potential short-term cardiovascular improvements, but current evidence supporting CR as a standalone, long-term therapy for obesity or heart disease remains limited and inconclusive [73].

### 3.4. Mediterranean Diet

While the exact components of an MD are up for debate, a typical MD includes whole grains, lean red meat, plenty of plant-based foods, moderate levels of fish and poultry, and pulses—with unrestricted olive oil serving as a key source of healthy monounsaturated fatty acids (MUFAs) [74]. Thirteen clinical studies (or 72%) out of a total of 7186 participants indicated that central obesity significantly decreased after adopting an MD. The studies included in this review examined adults aged 18–80 years, with most samples ranging between 30 and 65 years. Comorbidities such as obesity, metabolic syndrome, diabetes mellitus, and cardiovascular risk were present in nearly all studies, while some included only healthy participants. Several studies focused exclusively on men or women, though menopause status was not specified [75].

Recent clinical evidence demonstrates that a hypocaloric MD, characterized by low sodium and high potassium intake, confers significant cardiometabolic benefits in individuals with overweight or obesity and elevated blood pressure. Over a 3-month period, MD adherence led to substantial reductions in body weight, waist circumference, fat mass, and 24 h ambulatory systolic and diastolic blood pressure. Improvements were also observed in lipid profiles, insulin sensitivity, and overall body composition. These effects were comparable to those achieved with a ketogenic diet, underscoring the MD’s efficacy despite its lower protein and fat content. Notably, changes in the fat-to-lean mass ratio correlated with blood pressure improvements, suggesting a direct link between body composition and cardiovascular outcomes [76].

A comprehensive meta-analysis of 26 randomized controlled trials (N = 10,352) found that MD interventions led to significant reductions in body mass index, waist circumference, triglyceride levels, and fatty liver index among overweight or obese adults. However, improvements in other cardiovascular risk markers were negligible, and only one trial (i.e., PREDIMED) demonstrated reduced cardiovascular event incidence. Despite these metabolic benefits, the quality of evidence was generally low due to study limitations and risk of bias [77].

The MD may contribute to modest weight loss when combined with CR and physical activity and has shown benefits in reducing dyslipidemia. Its rich composition of monounsaturated fats, polyphenols, and dietary fiber is linked to improved lipid profiles and reduced inflammatory markers [78].

The MD is associated with multiple health benefits, particularly in reducing cardiovascular risk and managing obesity. It promotes weight loss and reduction in central adiposity, especially when combined with CR and physical activity. Greater adherence to the MD is linked to lower weight gain over time and a decreased risk of developing overweight or obesity. In individuals with obesity, the MD improves adipose tissue function by increasing small adipocytes, enhancing angiogenesis, and reducing inflammation, which supports metabolic health. The MD positively influences cardiovascular risk factors by improving lipid profiles, reducing blood pressure, decreasing oxidative stress, and lowering chronic low-grade inflammation. These effects contribute to reduced endothelial dysfunction and prothrombotic states, lowering the risk of coronary heart disease and stroke. Moreover, the MD is associated with improved insulin sensitivity, which plays a key role in preventing type 2 diabetes and metabolic syndrome, both conditions closely related to obesity. Although the MD contains a relatively high fat content, mainly from olive oil and nuts, long-term adherence does not lead to weight gain. Instead, it supports favorable changes in body composition and cardiovascular health. Thus, the MD represents an effective dietary strategy to prevent and manage obesity and cardiovascular diseases through multiple synergistic mechanisms [79].

Several RCTs have compared the MD with other eating plans, including those proposed by the American Diabetes Association, LCDs, and low-fat diets. The five RCTs included in this systematic review enrolled 998 overweight or obese participants, with a mean age range of 44 to 67 years. Most studies included individuals with significant comorbidities, such as type 2 diabetes, coronary heart disease, or recent myocardial infarction, while only one trial involved healthy overweight subjects. Physical activity was not used as a differential intervention, as co-interventions were applied equally across groups. The gender distribution varied: two studies had predominantly male populations (74–86%) and one study included mostly female participants (90.1%), while the others had a balanced gender distribution. The menopausal status of female participants was not reported. After 12 months or more, participants following a low-fat diet lost more weight than those on the MD (ranging from 2.9 to −5.0 kg vs. −4.1 to −10.1 kg). However, both diets achieved similar weight reductions (ranging from −4.7 to −7.7 kg vs. −4.1 to −10.1 kg). Improvement in other cardiovascular risk factors, such as lipids and blood pressure, was also observed with the MD, which was comparable to the effects of the comparator diets. The study suggests that, when compared to other diet plans, the MD helps overweight or obese individuals lose weight and reduce cardiovascular risk factors [80].

A randomized crossover trial comparing a low-calorie MD with a low-calorie lacto-ovo vegetarian diet (Vd) in overweight individuals further highlights distinct cardiovascular benefits despite similar weight loss outcomes. The study included 118 clinically healthy adults aged 18 to 75 years (median age: 50), with 78% female participants. Although menopausal status was not specified, the high proportion of women and the inclusion of individuals up to age 75 suggest that postmenopausal women were likely represented. Baseline assessments included self-reported physical activity, categorized qualitatively from absent/light to moderate, and participants were instructed to maintain their usual activity levels. Comorbidities were screened at baseline, and only participants with low-to-moderate cardiovascular risk and no serious illnesses or medication use were included. Both diets resulted in comparable reductions in body weight, BMI, and fat mass after three months. However, the Vd was more effective in lowering LDL cholesterol, whereas the MD demonstrated a greater reduction in triglyceride levels. Additionally, only the MD group showed a significant decrease in the inflammatory marker IL-17. These findings suggest that while both dietary patterns support weight loss, the MD may provide superior improvements in specific cardiovascular risk factors, potentially due to its unique composition rich in monounsaturated fats, fiber, and phytonutrients [81].

### 3.5. Ketogenic Diet

KDs are characterized by a significantly reduced carbohydrate intake (<30 g/day) and a consistent protein ratio (1.0–1.2 g/kg of fat-free mass or 1.2–1.5 g/kg of ideal body weight) [82]. The KD has been increasingly recognized not only for its effects on lipid metabolism, but also for its influence on bile acid signaling pathways. Recent findings suggest that changes in bile acid circulation and composition, particularly involving the farnesoid X receptor and Takeda G protein-coupled receptor 5, play a pivotal role in modulating energy expenditure, inflammation, and lipid homeostasis in high-fat-diet contexts [83].

Metabolic syndrome, obesity, cancer, polycystic ovary syndrome, epilepsy, neurological diseases, and type 2 diabetes mellitus are among the numerous medical conditions that frequently utilize these therapeutic strategies. A clinical study suggests that a KD can have beneficial long-term effects on obese individuals, particularly in reducing body weight, BMI, and blood glucose levels. Additionally, it improves lipid profiles by significantly increasing HDL cholesterol and reducing LDL cholesterol and triglycerides. The findings also indicate that a KD is safe for extended periods, with no major adverse effects on kidney function as measured by creatinine and urea levels. These results support the potential of the KD as a viable and effective dietary intervention for obesity management and metabolic health improvement [82]. However, there are also potential risks regarding this type of diet. One of the key cardiovascular concerns of the KD is its potential to increase LDL cholesterol and triglyceride levels. These lipid changes may offset the diet’s intended metabolic benefits [84]. Prolonged adherence to the KD may result in complications such as fat accumulation in the liver, low protein levels, electrolyte imbalances, and inadequate vitamin intake. Despite its well-documented advantages, maintaining this dietary regimen over the long term remains a significant challenge [85].

In children with epilepsy, where the KD is part of the overall management, notable adverse effects include electrolyte imbalances, dehydration, and hepatic issues, suggesting possible systemic strain. Nonetheless, these effects may be influenced by underlying health conditions and the specific formulation used in epilepsy treatment [86].

Scientific evidence indicates that the KD can significantly reduce body weight, improve glycemic control, and favorably modify lipid profiles by increasing HDL cholesterol and lowering triglycerides. However, the impact on LDL cholesterol is inconsistent, with some studies showing increases, raising concerns about long-term cardiovascular risk. Notably, the KD induces nutritional ketosis, where ketone bodies exert potent anti-inflammatory and cardioprotective effects by inhibiting pro-inflammatory pathways and improving endothelial function. These mechanisms may contribute to reductions in blood pressure and overall cardiovascular risk beyond weight loss alone. Despite encouraging findings, heterogeneity in study designs and variable effects on lipid fractions highlight the need for more rigorous, long-term research to clarify the KD’s role in obesity management and cardiovascular prevention [87].

The KD offers several short-term benefits for obesity and cardiovascular risk factors. It significantly reduces body weight, BMI, fat mass, and waist circumference, particularly during the initial ketosis phase (up to 12 weeks). Improvements in lipid profile include decreased triglycerides and increased HDL cholesterol, although the effects on LDL cholesterol vary. The diet also supports better glycemic control, lowering fasting glucose and HbA1c levels, especially in diabetic patients within 3 to 6 months of intervention. Blood pressure reductions have been documented, likely due to weight loss and increased diuresis associated with ketosis. Importantly, studies show that the KD is generally safe in the short term, with no major adverse effects on kidney function. These metabolic improvements suggest that the KD can be an effective, supervised dietary approach for rapid weight loss and short-term cardiovascular risk reduction in obese individuals, though long-term benefits require further research [88].

A meta-analysis of 27 randomized controlled trials involving 1278 participants showed that KDs significantly reduce body weight, BMI, triglycerides, blood glucose, insulin, and diastolic blood pressure, suggesting metabolic benefits for obesity and cardiovascular risk. However, KDs also increase total cholesterol and LDL cholesterol, potentially raising cardiovascular risk. These mixed effects warrant cautious interpretation and further research to clarify the long-term safety and efficacy of KDs in managing obesity and cardiovascular health [89].

Furthermore, medical reports indicate that individuals with obesity can reduce their diastolic and systolic blood pressure using a KD over 48 weeks compared to a low-fat diet supplemented with orlistat [90]. A large NHANES study found that KD adherence was linked to reduced all-cause mortality risk, but there was no significant impact on cardiovascular mortality in US adults [91].

### 3.6. DASH Diet

The DASH diet is designed to lower blood pressure by emphasizing low-fat dairy, fruits, and vegetables and reducing sugar and red meat. It is low in sodium, total fat, saturated fat, and cholesterol, while being rich in calcium, potassium, and magnesium. Beyond blood pressure reduction, the DASH diet also helps reduce the risk of overweight and obesity, lower total and LDL cholesterol levels, and regulate glucose and insulin imbalances. While its effects on triglycerides and HDL cholesterol are less significant, its nutrient-rich components support overall metabolic health [92].

The stratification of individuals with obesity into metabolically healthy obese (MHO) and metabolically unhealthy obese (MUHO) phenotypes has been established to differentiate those who, despite excess adiposity, lack overt cardiometabolic dysfunction from those who manifest clinically relevant metabolic impairments [93].

A population-based prospective study suggests that adherence to the DASH diet may help reduce the likelihood of developing metabolic unhealthy obesity (MUHO) in overweight or obese individuals [94]. Obesity phenotypes, including MUHO and metabolically healthy obesity (MHO), were classified based on the Joint Interim Statement criteria for metabolic syndrome [95], which rely on clinical and biochemical cardiometabolic risk markers rather than insulin resistance or inflammatory profiles. Individuals were designated as MUHO if they met at least two of the following: elevated waist circumference (≥91 cm in women, ≥89 cm in men), fasting plasma glucose ≥100 mg/dL or antidiabetic treatment, triglycerides ≥150 mg/dL or lipid-lowering medication, low HDL-C (<50 mg/dL in women, <40 mg/dL in men) or drug therapy, and elevated blood pressure (systolic blood pressure ≥130 mmHg or diastolic blood pressure ≥85 mmHg) or use of antihypertensives. Participants with no more than one abnormality were considered MHO. By improving dietary habits, particularly through the DASH diet, individuals may lower their risk of metabolic abnormalities associated with obesity. These findings highlight the potential clinical implications of the DASH diet as a preventive approach to mitigate the metabolic complications commonly seen in obesity, offering a promising dietary strategy for improving metabolic health in at-risk populations [94].

Various dietary interventions for weight loss differ not only in their calorie restrictions but also in their macronutrient composition and timing, each producing distinct metabolic effects. CR diets effectively promote general weight loss, while very-low-calorie diets are typically reserved for severe obesity and metabolic disorders like type 2 diabetes. Timing-based approaches such as intermittent fasting and meal timing adjustments can improve insulin sensitivity and support metabolic flexibility by optimizing circadian rhythms. Macronutrient-focused diets show more nuanced impacts: low-fat diets are generally recommended for heart health and cholesterol management, whereas low-carbohydrate diets and KDs often lead to rapid weight loss and improved glycemic control but may raise concerns about long-term cardiovascular risk due to inconsistent effects on LDL cholesterol. High-protein diets help sustain weight loss and reduce hunger, supporting better adherence. The MD stands out for its balanced nutrient profile and the strong evidence that it promotes cardiometabolic health and long-term metabolic stability [96,97].

### 3.7. Impact of Weight Loss on Cardiovascular Risk

It has been observed in a medical study that weight loss in individuals with type 2 diabetes is strongly linked to improvements in cardiovascular risk factors. After one year, reductions in body fat resulted in better glucose tolerance, higher HDL levels, lower triglycerides, and improved blood pressure. Greater weight loss (10–15%) led to even more significant health improvements [98].

A study found that intentional weight loss in overweight individuals with type 2 diabetes can reduce overall mortality by 16% and lower CVD risk. Bariatric surgery reduces CVD risk by 32%, while dietary weight loss reduces it by 21%. Weight loss through a reduced-carbohydrate, higher-protein diet improves risk factors like blood pressure and triglycerides, though it does not directly reduce CVD events or total mortality [99]. Furthermore, a trial involving 2041 individuals found that weight loss resulted in significant improvements in cardiovascular risk factors. For each kilogram of weight lost, reductions of 0.4 mmHg in diastolic blood pressure and 0.5 mmHg in systolic blood pressure were observed, along with a 0.2 mmol/mol drop in HbA1c. No impact on plasma lipids was noted. These findings suggest that weight loss, particularly through community weight-loss programs, can improve glucose regulation and blood pressure, especially in high-risk individuals [100].

Behavioral weight reduction programs for overweight or obese individuals resulted in significant improvements in cardiovascular risk factors. Participants who lost 5–10% of their body weight experienced reductions in triglycerides, total cholesterol, and LDL cholesterol. Those who lost more than 10% saw even greater improvements in these markers, except HDL cholesterol, highlighting the benefits of higher weight loss in reducing cardiovascular risks [101].

## 4. Molecular Pathways at the Interface of Obesity, Diet, and Cardiovascular Diseases

Elevated concentrations of free fatty acids and triglycerides commonly observed in obesity contribute to abnormal lipid deposition in non-adipose organs, notably the liver, myocardium, and skeletal muscles. This ectopic fat storage is a key driver of insulin resistance, elevated blood pressure, type 2 diabetes, metabolic syndrome, atherogenesis, and cardiovascular complications. In the myocardial context, the combined effects of obesity and type 2 diabetes disrupt normal metabolic pathways and shift cardiac energy substrate preferences, while concurrently increasing oxidative burden and inflammatory signaling. These disturbances collectively foster fibrotic remodeling and impair cardiac performance. Peroxisome proliferator-activated receptors (PPARs) are nuclear regulators that modulate glucose and lipid homeostasis, exert anti-inflammatory and antioxidative effects, and enhance insulin responsiveness and lipid clearance [45].

Poor nutritional habits represent a major contributor to both excess body weight and the development of cardiovascular disorders [102]. Consequently, adopting dietary strategies aimed at optimizing the management of both obesity and cardiovascular disease, alongside elucidating the molecular mechanisms underpinning the diet–obesity–CVD axis, becomes critically important.

### 4.1. Dietary Patterns and Mechanisms of Function Against Obesity and CVD

Numerous dietary approaches have been proposed for managing obesity and cardiovascular disease, each demonstrating specific benefits and limitations. The following section critically reviews the most studied dietary patterns in terms of their impact on these conditions, followed by a detailed exploration of the mechanisms through which they exert their effects.

Historically, diets low in fat have been advocated for controlling body weight and promoting cardiovascular well-being. These nutritional regimens typically yield modest reductions in body mass and enhance lipid metrics, notably through decreases in total cholesterol and LDL concentrations. Nonetheless, their long-term impact on weight stabilization and CVD prevention remains inconclusive and continues to be debated in the current literature [103]. Limiting dietary fat intake reduces the caloric density of meals, thereby supporting energy intake reduction and subsequent weight loss. Additionally, restricting saturated fat lowers circulating LDL by diminishing hepatic cholesterol absorption and influencing LDL receptor dynamics. Furthermore, low-fat diets may positively affect endothelial performance by mitigating systemic inflammation and oxidative damage [104,105].

Nutritional plans with reduced carbohydrate contents have seen increased use for weight control and metabolic improvement. These diets are associated with marked reductions in weight, BMI, central adiposity, triglycerides, and systolic and diastolic blood pressure. While favorable modifications in cardiovascular risk indicators have been observed, further longitudinal studies are essential to confirm durable benefits [66,106]. CR enhances insulin responsiveness, promoting fatty acid mobilization and oxidation, which collectively reduce adiposity and augment insulin sensitivity [107,108]. Moreover, such diets generally increase HDL-C and lower triglycerides through enhanced hepatic lipid oxidation. Nonetheless, a concurrent rise in LDL may occur due to elevated endogenous cholesterol synthesis and modifications in LDL particle characteristics [109,110].

Diets characterized by reduced total caloric intake have proven to be effective in promoting weight reduction and cardiovascular risk mitigation. These regimens decrease body weight and arterial pressure and improve lipid parameters. However, compliance often declines over time, and regaining lost weight is frequently reported [111,112]. A hypocaloric intake generates an energy deficit, leading to fat mass loss and lower adipose tissue-derived inflammatory markers, such as IL-6 and TNF-α. This fosters improvements in insulin sensitivity and vascular function. CR is also known to enhance mitochondrial activity and decrease oxidative burden, contributing further to cardiovascular health [113,114].

The MD is frequently associated with diminished risk for both obesity and cardiovascular events. This dietary model, emphasizing fruits, vegetables, legumes, whole grains, nuts, olive oil, and moderate fish and poultry intake, is consistently linked with reductions in BMI, central adiposity, and better lipid regulation [79]. Furthermore, the diet’s high fiber content promotes a healthy gut microbiome and influences short-chain fatty acid production, which in turn stimulates AMPK activity [115].

Several pathways explain the MD’s health benefits. Its high content of polyphenols and monounsaturated fats counteracts inflammation and oxidative stress through inhibition of NF-κB, MAPKs, and PI3K/Akt signaling, while enhancing antioxidant enzyme systems like SOD, CAT [116], and glutathione peroxidase, an enzyme with implications for the metabolic syndrome [117]. This diet also enhances LDL receptor activity, facilitating LDL clearance, and impairs cholesterol absorption, aided by phytosterols, thereby reducing total cholesterol, LDL, and ApoB-100 [118]. Nitric oxide availability is increased, supporting endothelial function and lowering blood pressure [119]. Moreover, the intake of fiber-rich, plant-based foods boosts beneficial microbial populations, raising short-chain fatty acid levels, which enhance both metabolic and inflammatory parameters [119,120].

Emerging evidence highlights the NLRP3 inflammasome as a key mediator in metabolic inflammation, contributing to insulin resistance and β-cell dysfunction. The dietary composition of the MD plays a modulatory role in this pathway [121]. Studies show that certain polyphenols, including those from raspberry, grape seed, and jaboticaba, can inhibit NLRP3 inflammasome activation in obesity-related models. This leads to reduced levels of caspase-1, IL-1β, and IL-18, especially in liver, kidney, and intestinal tissues. These changes are linked to improved insulin sensitivity, lower body weight, and better glucose control. Some effects were enhanced when combined with probiotics or unsaturated fats, suggesting a synergistic anti-inflammatory role of diet in modulating innate immune responses via NLRP3 [122,123].

The KD, known for its high-fat and minimal carbohydrate structure, shows significant efficacy in short-term weight loss and glycemic management [124]. However, concerns persist regarding its prolonged influence on lipid profiles, particularly elevated LDL [125]. By depriving the body of carbohydrates, the KD induces a metabolic transition favoring ketone body generation and fatty acid oxidation. In obese individuals, the KD reliably reduces BMI, abdominal fat, hypertension, and insulin resistance. Appetite suppression, partly driven by ketone bodies such as β-hydroxybutyrate (β-HB), contributes to reduced energy intake [126,127]. β-HB, raised by KDs, functions beyond energy supply by influencing metabolism via inhibition of HDACs and activation of specific receptors. Its breakdown elevates acetyl-CoA, reduces succinyl-CoA, and maintains NAD+ levels, which supports protein acetylation and mitochondrial activity. These changes enhance insulin response and glucose processing and lower oxidative damage. The KD also suppresses insulin and mTOR pathways, stimulates AMPK, and increases antioxidant gene expression, fostering metabolic adaptability. β-HB’s molecular effects help improve obesity and cardiovascular health by modulating gene regulation, metabolic pathways, and inflammation [128].

Additionally, the KD triggers metabolic shifts that raise energy expenditure due to gluconeogenesis and protein turnover. Independent of weight loss, this diet enhances insulin function and lipid profiles [126,127]. In cardiovascular contexts, the KD may enhance cardiac energetics and reverse adverse remodeling, partly through PPARα activation and ROS reduction. Nonetheless, risks such as myocardial fibrosis, apoptosis, prolonged QT intervals, and mitochondrial dysfunction via Sirt7 overexpression have been observed. Elevated β-HB concentrations have also been correlated with increased cardiovascular risk in some cohorts, underscoring the need for clinical caution in CVD patients [87,126].

The DASH dietary model was developed to target blood pressure control and cardiovascular risk reduction. Centered on high consumption of fruits, vegetables, whole grains, lean protein, and low-fat dairy with restricted sodium, DASH is linked to reductions in weight, blood pressure, and favorable shifts in lipid markers [129,130]. The DASH diet benefits cardiovascular health through several mechanisms. Elevated intakes of potassium, magnesium, and calcium facilitate vasodilation and promote natriuresis, thereby decreasing blood pressure [129]. Enhanced production of nitric oxide leads to improved endothelial reactivity [131]. Additionally, reducing saturated fats results in lower LDL and VLDL levels [132], while high dietary fiber improves glucose metabolism and insulin effectiveness [133].

### 4.2. Integrated Mechanistics of Dietary Interventions

While differing in structure and macronutrient composition, the dietary strategies reviewed commonly influence core physiological mechanisms, including energy balance modulation, insulin action enhancement, and inflammation control. CR, an inherent component of low-calorie, low-fat diets and often KDs, stimulates mitochondrial biogenesis and lowers oxidative damage through AMPK and sirtuin activation [134]. Both low-carbohydrate diets and KDs shift substrate utilization toward lipids and ketones, boosting adiponectin levels and reducing insulin, which enhances metabolic profiles [135,136]. Nonetheless, these approaches can elevate LDL due to upregulation of hepatic cholesterol synthesis and altered lipoprotein composition [137]. Conversely, MD and DASH diets, though not macronutrient-restrictive, exert anti-inflammatory effects via abundant polyphenols and unsaturated fats, which suppress NF-κB and activate Nrf2 pathways, also promoting nitric oxide-mediated vasodilation [138]. Unlike long-term ketogenic or low-carb regimens, which may impair endothelial responsiveness, these two dietary models improve vascular integrity by supporting antioxidant enzyme expression and lipid metabolism [139]. Additionally, fiber-rich diets like DASH and the MD influence the gut microbiome favorably, increasing short-chain fatty acid production and supporting systemic metabolic regulation [129,140]. Thus, although all regimens impact cardiovascular and metabolic health, they do so through distinctly varying biochemical and physiological channels.

### 4.3. Comparative Analysis of Dietary Patterns Regarding Obesity and Cardiovascular Health

Nutrition plays a foundational role in the prevention and management of both obesity and cardiovascular disease. Among the spectrum of dietary regimens, the MD and DASH diets consistently produce the most favorable outcomes. The Mediterranean approach, rich in plant-based foods and healthy fats, is well documented for its effects in lowering body weight and arterial pressure and improving cholesterol profiles, notably through reductions in total cholesterol and LDL [141]. Similarly, the DASH model promotes cardiovascular benefits via nutrient-dense, low-sodium components and has demonstrated robust efficacy in lowering blood pressure and improving lipid health [129].

On the other hand, carbohydrate-restricted diets, including ketogenic variations, are often superior for initial weight loss compared to low-fat diets [57]. However, caution is warranted regarding potential long-term elevations in LDL, despite increases in HDL-C and reductions in triglycerides [137]. Low-fat regimens have modest benefits on lipid parameters and body weight but are generally considered less impactful than other models [141]. CR diets, focusing on reducing overall energy intake, have demonstrated positive effects on weight loss and metabolic health [142]. Studies like the CALERIE trials have shown that sustained calorie restriction can lead to improvements in body composition, insulin sensitivity, and lipid profiles [143].

While multiple dietary patterns yield positive effects on metabolic and cardiovascular outcomes, the MD and DASH diets offer the most sustainable and well-rounded benefits. Diets like ketogenic [144] or calorie-restricted approaches may yield more rapid short-term results but should be monitored closely for long-term safety and adherence concerns.

## 5. Conclusions

CVDs are leading global causes of mortality, and understanding the molecular mechanisms linking them to obesity and diet can significantly improve patient outcomes. The molecular metabolic pathways involved in inflammation, insulin resistance, and cardiovascular dysfunction are complex and can also be modulated by nutritional factors included in diet patterns. Future randomized trials will further clarify the optimal dietary strategies for CVD patients, aiding healthcare providers in delivering tailored recommendations. Effective collaboration among physicians, nursing staff, and dietitians is essential for thorough nutritional assessments and patient care. Integrating dietitians into primary care teams ensures more precise dietary guidance, as intensive nutritional counseling has demonstrated strong benefits in correcting imbalances and improving health outcomes. Standardized, continuous nutritional interventions should become an integral part of cardiovascular care protocols.

## Abbreviations

The following abbreviations are used in this manuscript:

AChID	Acetylcholine-induced dilation
AF	Atrial fibrillation
AMPK	Adenosine monophosphate-activated protein kinase
Ang II	Angiotensin II
AT1r	Angiotensin II type 1 receptor
BMI	Body mass index
CAD	Coronary artery disease
CHD	Coronary heart disease
CR	Caloric restriction
CVD	Cardiovascular disease
DASH	Dietary Approaches to Stop Hypertension
Dectin-1	Pattern recognition receptor Dectin-1
ERK	Extracellular signal-regulated kinases
FGF-21	Fibroblast growth factor 21
FID	Flow-induced dilation
GDF-15	Growth differentiation factor 15
HbA1c	Hemoglobin A1c
HDL	High-density lipoprotein
HP-IF	High-protein intermittent fasting
IFCR	Intermittent fasting combined with caloric restriction
IL	Interleukin
IRS	Insulin receptor substrate
JAKs	Janus kinases
LCD	Low-carbohydrate diet
LDL	Low-density lipoprotein
LFWM	Low-fat weight maintenance
LFWL	Low-fat weight loss
MAPKs	Mitogen-activated protein kinases
MCP	Monocyte chemoattractant protein
MD	Mediterranean diet
mTOR	Mechanistic target of rapamycin
MUHO	Metabolic unhealthy obesity
L-NAME	Nω-nitro-l-arginine methyl ester
NADPH	Nicotinamide adenine dinucleotide phosphate
NF-κB	Nuclear factor kappa-light-chain-enhancer of activated B cells
NO	Nitric oxide
Ox-LDL	Oxidized low-density lipoprotein
PI3K	Phosphatidylinositol 3-kinase
PPARs	Peroxisome proliferator-activated receptors
RAS	Renin–angiotensin system
ROS	Reactive oxygen species
QRISK	10-year cardiovascular risk
S6K1	Ribosomal protein S6 kinase 1
SCFAs	Short-chain fatty acids
SNS	Sympathetic nervous system
STAT	Signal transducer and activator of transcription
TGs	Triglycerides
TMAO	Trimethylamine N-oxide
TNF-α	Tumor necrosis factor-alpha
Vd	Vegetarian diet
VLDL1-TGs	Very-low-density lipoprotein triglycerides
WC	Waist circumference
WHR	Waist-to-hip ratio
WHtR	Waist-to-height ratio
